# Insights on altered mitochondrial function and dynamics in the pathogenesis of neurodegeneration

**DOI:** 10.1186/2047-9158-2-12

**Published:** 2013-05-27

**Authors:** Joseph McInnes

**Affiliations:** 1 School of Engineering and Science, Research Center MOLIFE–Molecular Life Science, Jacobs University Bremen, Campus Ring 1, 28759 Bremen, Germany

**Keywords:** Mitochondria, Neurodegeneration, Parkinson’s, Alzheimer’s, Charcot-Marie-Tooth

## Abstract

In neurons, mitochondria are enriched to provide energy and calcium buffering required for synaptic transmission. Additionally, mitochondria localize to the synapse, where they are critical for the mobilization of reserve pool vesicles and for neurotransmitter release. Previously, functional defects in mitochondria were considered to be downstream effects of neurodegenerative diseases. However, more recent findings suggest mitochondria may serve as key mediators in the onset and progression of some types of neurodegeneration. In this review, we explore the possible roles of altered mitochondrial function and dynamics in the pathogenesis of neurodegenerative disorders, with a particular focus on Alzheimer’s disease (AD) and Parkinson’s disease (PD), which have highlighted the important role of mitochondria in neurodegeneration. While inheritable diseases like Charcot-Marie-Tooth disease type 2A are concretely linked to gene mutations affecting mitochondrial function, the cause of mitochondrial dysfunction in primarily sporadic diseases such as AD and PD is less clear. Neuronal death in PD is associated with defects in mitochondrial function and dynamics arising from mutations in proteins affecting these processes, including α-synuclein, DJ-1, LRRK2, Parkin and Pink1. In the case of AD, however, the connection between mitochondria and the onset of neurodegeneration has been less clear. Recent findings, however, have implicated altered function of ER-mitochondria contact sites and amyloid beta- and/or tau-induced defects in mitochondrial function and dynamics in the pathogenesis of AD, suggesting that mitochondrial defects may act as key mediators in the pathogenesis of AD as well. With recent findings at hand, it may be postulated that defects in mitochondrial processes comprise key events in the onset of neurodegeneration.

## Introduction

In addition to providing the large amount of energy required for synaptic communication in the form of adenosine triphosphate (ATP), mitochondria also participate in a wide variety of other neuronal processes including calcium buffering, intracellular signaling, lipid flux, and mediate contact sites between mitochondria and the endoplasmic reticulum (ER). New findings have complicated our view of the mitochondrion by the elucidation of new roles for mitochondria in cellular physiology, which extend far beyond the traditional role of ATP synthesis. The growing number of of identified processes in which mitochondria play a role has also led to new connections between mitochondrial dysfunction and various pathologies in humans [1].

A variety of factors contribute to mitochondrial health and fitness. Human mitochondrial DNA (mtDNA) encodes 13 proteins, which are essential for the assembly and function of the mitochondrial respiratory complexes. Given the small size of the mtDNA molecule, and its high susceptibility to mutations due to close proximity to reactive oxygen species (ROS, a byproduct of respiration), mtDNA maintenance and repair mechanisms are critical for maintaining mitochondrial function [2]. In neurons, defects in mtDNA damage repair lead to the accumulation of deleterious mutations, resulting in decreased mitochondrial respiratory activity and eventually neuronal death [3]. In addition to mtDNA, the nuclear DNA (nDNA) encodes over 1000 mitochondrial proteins, which are required for various metabolic and functional processes. Therefore, nDNA maintenance and repair mechanisms in the nucleus also play an important role in maintaining mitochondrial fitness, as the mutation of nDNA genes encoding mitochondrial proteins can lead to pathology [4].

An important factor contributing to mitochondrial health is the dynamic balance of fission and fusion. Since mitochondria cannot be synthesized *de novo*, the biogenesis of new mitochondria must begin from existing mitochondria. Mitochondrial fission is the process whereby one mitochondrion physically separates into two independent mitochondrial objects. To carry out fission, the mitochondrial-localized Mff protein recruits the cytosolic dynamin-related Drp1 to the mitochondrial outer membrane [5]. Once recruited to the membrane, Drp1 provides the mechanical force to pinch the membrane to cause fission [6]. Fission is not only important for increasing the number of mitochondria within a cell, but also for the disposal of debris by mitophagy-mediated pathways, and is therefore essential for maintaining mitochondrial fitness [7,8]. Mitochondrial stress induces fission, and since mitochondrial dysfunction is concomitant with neuronal death, mitochondrial fragmentation due to excessive fission is a hallmark of some neurodegenerative diseases [9]. Moreover, it has been shown that normal levels of mitochondrial fission are required for the mobilization of synaptic vesicles from reserve pools for release into the synaptic cleft, and neurons deficient in Drp1 fail to maintain normal neurotransmission following stimulation [10].

The opposite process of mitochondrial fission is fusion, whereby two mitochondria physically combine to become one single mitochondrion with a continuous membrane. The GTPases Mfn1, Mfn2 and OPA1 are required for mitochondrial fusion, whereby the mitofusins Mfn1 and Mfn2 facilitate outer membrane fusion and OPA1 facilitates inner membrane fusion [11]. Mitochondria are constantly undergoing fusion and fission at various time points throughout their lifecycle, and this dynamic balance gives rise to the diverse mitochondrial morphology observed in cells [12]. A damaged mitochondrion may be rescued by its fusion with a healthy mitochondrion, thereby promoting health and function of the mitochondrial population to ensure cellular fitness [13].

Recent years of research have elucidated the role of interactions between the ER and mitochondria. ER-mitochondrial interactions and contact sites are important for processes including lipid modification and flux [14,15], calcium buffering [16], mitochondrial fission [17,18], and organelle inheritance [19,20]. In neurons, ER-mitochondria contact sites are particularly important as sites of calcium buffering, where ER and mitochondria coordinate the release of large amounts of calcium ions into the cytosol for intracellular signaling [21]. Notably, an increased concentration of calcium ions in mitochondria leads to an increase in respiratory complex activity and ATP production, providing an important link between intracellular signaling and mitochondrial respiration [22]. Importantly, ER-mitochondria contact sites mark sites of mitochondrial fission, as they play a role in recruiting Drp1 to Mff receptors, and also provide the initial mechanical force to constrict the membrane [17].

In neurodegeneration, mitochondrial dysfunction leads to widespread disturbances in processes critical for neuronal function, including coordinated calcium signaling and neurotransmitter release at the synapse. Though recent research has revealed more links between defects in specific mitochondrial processes and the progression of neurodegeneration, the exact executors (i.e. that which mediates the onset of pathogenesis and neurodegeneration) and pathomechanisms thereof remain poorly defined. Nonetheless, growing evidence suggests mitochondria play a key role in the execution of neurodegeneration, either due to defects in respiratory function or by interaction with other organelles, such as the ER or cytoskeleton. This review attempts to clarify connections between defects in mitochondrial processes and the execution of neurodegeneration, demonstrating that mitochondria are now entering the spotlight for investigating the pathological basis of neurodegenerative diseases.

### Parkinson’s disease

The hallmark characteristic of all adult-onset neurodegenerative disorders is the degeneration or death of neurons. In can be broadly stated that this is due to a breakdown in one or more cellular processes essential for fitness, often involving the loss of quality control and recycling mechanisms such as autophagy and the degradation of aggregated or unfolded proteins. Parkinson’s disease (PD) is a devastating neurodegenerative disorder affecting about 4% of seniors over 80 years old, with an average onset around age 60 [23]. While a small portion of PD cases is inherited or due to other known genetic factors, most cases of PD are sporadic. PD is characterized by death of the dopaminergenic neurons (up to 70% by the time of death) in the substantia nigra region of the brain. This resulting decrease in dopamine levels leads to motor disorders such as limb tremors, stiffness, slowness of movement, and postural instability. Additionally, there is no definitive test for the clinical diagnosis of PD and current treatment only aims to reduce the severity of symptoms [24].

Initially, the most long-standing hypothesis considered the primary executor of neuronal death in PD to be the formation of α-synuclein plaques, composed of aggregated and insoluble α-synuclein protein in Lewy bodies [25,26]. Though it was long believed that α-synuclein was the primary executor of PD, mitochondria were brought into the spotlight at the end of the 1990s when mutations in a protein named Parkin were identified in a Japanese PD patient [27]. In coming years, it would be described that Parkin is required for the degradation of poorly functioning mitochondria by mitophagy [7,28]. Soon after the discovery of *Parkin* mutations linked to PD, another key player, the PTEN-induced kinase 1 (Pink1), would be identified as mutated in PD patients [29]. Today, it is known that Pink1 acts upstream of Parkin, and that the interaction and/or function of these two proteins is altered in PD patients, commonly resulting from mutations in one or both genes in familial cases of PD [30]. Moreover, recent studies have found that α-synuclein itself can localize to mitochondria and modulate their function by down-regulating complex I activity in a dose-dependent manner [31], and results in mitochondrial fragmentation upon overexpression by promoting translocation of Drp1 to mitochondria [32].

The wealth of information gained from studying the roles of Pink1 and Parkin in PD has given rise to new hypotheses of how mitochondrial dysfunction may influence the pathogenesis of PD. Evidence suggests that in healthy cells, the Pink1/Parkin pathway may act to control for and clear dysfunctional mitochondria, characterized by high ROS levels and low membrane potential [13]. When a mitochondrion becomes dysfunctional, Pink1 attaches to the outer mitochondrial membrane, where it will recruit Parkin [33]. Upon recruitment of Parkin, mitochondria become ubiquitinated, leading to the induction of mitophagy [7]. Interestingly, the kinase activity of Pink1 does not act on Parkin, suggesting that there are other key mediators of mitochondrial function and quality control, the elucidation of which will likely prove fruitful in understanding and treating PD [34]. Moreover, major mitochondrial defects such as elevated oxidative stress and a 20-30% decrease in the activity of electron transport chain complexes are characteristic of PD patients [35,36]. Taken together, this data may suggest that in the case of PD, mutations in Pink1 and/or Parkin prevent the clearance of dysfunctional mitochondria; because mitochondrial function is critical for providing energy to the cell as well as for synaptic communication, this would ultimately lead to neuronal death.

It is important to note, however, that experiments investigating the role of Pink1/Parkin as possible mediators of mitochondrial function in PD have been limited by their cellular models. Notably, investigations into the possible functions of the Pink1/Parkin system in maintaining cellular fitness have been limited to cultured cells treated with the mitochondrial-uncoupling compound carbonyl cyanide *m*-chlorophenylhydrazone (CCCP), and there is currently little concrete evidence that the Pink1/Parkin pathway is active in healthy cells. Therefore, how mitochondrial fitness, the Pink1/Parkin pathway, and the onset of neurodegeneration in PD relate to one another *in vivo* remains to be concretely defined. Additionally, experiments using different model organisms have concluded with inconsistent results as to how mutations in Pink1 and/or Parkin may influence mitochondrial dynamics. In particular, while studies in *Drosophila* have shown that Pink1 deficiency leads to swollen, perinuclear mitochondria [37,38], Pink1 deficiency in mammalian cells leads to mitochondrial fragmentation [39,40]. Thus, the exact mechanism how Pink1 influences mitochondrial dynamics in humans remains undefined; nonetheless, it is evident that Parkin and Pink1 play an important role in regulating mitochondrial fission and fusion, and that this may contribute to overall neuronal fitness.

In addition to α-synuclein, Parkin and Pink1, mutated forms of the proteins DJ-1 and LRRK2, as are found in specific forms of familial PD, have also recently been implicated as modifiers of mitochondrial dynamics. Mutations in the leucine-rich repeat kinase 2 (LRRK2) are commonly found in both familial (autosomal-dominant) and sporadic cases of PD. LRRK2 is a large multi-domain protein kinase of unknown function, which localizes to the cytosol and associates with the mitochondrial outer membrane [41]. Recent data has shown that overexpression of wild-type LRRK2, as well as the expression of mutated LRRK2 isoforms present in autosomal-dominant PD patients, lead to mitochondrial fragmentation by direct interaction with Drp1 [42]. Moreover, mutations in the DJ-1 protein, as found in cases of autosomal-recessive PD, result in increased Drp1 levels and increased mitochondrial fragmentation [43]. Thus, altered mitochondrial dynamics, and in particular mitochondrial fragmentation, is a notable characteristic in many forms of PD.

In summary, PD pathogenesis has been closely associated with defects in mitophagy and altered mitochondrial dynamics, and it is likely a combination of these defects which leads to neuronal dysfunction during the pathogenesis of PD. Current avenues of research are focusing on the genetic factors affecting mitochondrial function, as well as modifiers and possible rescue mechanisms thereof. Currently, efforts to develop therapies for treating PD focus on either reducing protein plaques or alleviating mitochondrial dysfunction, for example by inducing expression of mitochondrial neuronal uncoupling proteins [44] or bypassing respiratory complex defects to restore normal ATP production. Further research into the Pink1/Parkin pathway of mitophagy will also likely be fruitful in identifying new proteins involved in this pathway, thereby providing new opportunities for therapeutic targets.

### Alzheimer’s disease

Like Parkinson’s, Alzheimer’s disease (AD) is another devastating neurodegenerative disorder, affecting an astounding ~6% of North Americans and Western Europeans above the age of 60, and over 25% above the age of 85 [45]. The majority of AD cases are sporadic, though an estimated 1-5% of patients carry genetic mutations that are thought to contribute to the onset of AD. AD patients present symptoms of dementia, including loss of long-term memory, confusion, mood swings, and loss of language skills [46]. In the case of AD, there are currently multiple well-supported hypotheses as to its causes and executors. The hallmark of AD is the death of neurons in the cortex and hippocampus of the brain, and a single executor of this neuronal death remains elusive [47].

Similar to PD, the formation of protein plaques is characteristic of AD. One hypothesis holds that the aggregation of amyloid β-peptide (Aβ) (involved in synaptic formation and repair) into plaques is the executor of AD [48], which has also been genetically linked to familial AD cases in which mutations in the *APP* gene lead to increased amounts of Aβ [49]. The *APP* gene encodes the amyloid precursor protein (APP), which is processed by α-, β- and/or γ-secretases to generate Aβ. In some AD patients, one or more mutations near or within the Aβ-encoding region of *APP* increase the affinity of APP for β- and γ-secretase, thereby producing an estimated six-fold increase in Aβ [50,51]. Additionally, some mutations within the Aβ-encoding region also increase the affinity of Aβ to self-aggregate into plaques, which are neurotoxic [52]. Importantly, Aβ accumulates at mitochondria and inhibits respiratory complex IV, increasing ROS production and thereby increasing the frequency of mtDNA mutations [53]. Moreover, the studies of Wang et al. have found that Aβ itself alters mitochondrial dynamics by regulating the activities of proteins essential for fission and fusion, such as Drp1, which can lead to mitochondrial fragmentation [54-56]. These studies have been further supported by findings that Aβ interacts with Drp1, and that APP leads to widespread defects in mitochondrial biogenesis, transport and synaptic activity [57,58]. As discussed further below, altered mitochondrial dynamics may comprise a key event in AD pathogenesis.

A second hypothesis has become popular in recent years, which holds that hyperphosphorylation of the microtubule-stabilizing tau protein, sporadically or by genetic mutation [59], leads to its aggregation and therefore the formation of neurofibrillary tangles which act as executors of AD [60,61]. Tau is a microtubule-associated protein, which binds microtubules in an unphosphorylated state and stabilizes them. When mutations in the tau-encoding *MAPT* gene lead to hyperphosphorylation of tau, there is a two-fold effect. First, hyperphosphorylated tau is more likely to aggregate and form neurotoxic protein plaques, and does so in tauopathies. Second, decreased tau binding to microtubules decreases their stability. Mitochondria are transported to the synapse along the axon on linear microtubules by the action of a kinesin KIF5 motor protein [62], where they are critical for synaptic transmission [63]. Thus, the destabilization of microtubules is likely to affect mitochondrial transport to the axon, and thereby impair synaptic communication.

In the case of PD, recent findings have directly linked some PD-associated mutated genes with defects in mitochondrial function and/or dynamics. However, as mutations in genes relevant to AD are primarily either in genes encoding APP or tau, the role of mitochondria in the pathogenesis of AD is less clear. Nonetheless, research in the last year has renewed attention in the role of mitochondria in the pathogenesis of AD with the findings that mutations in APP and tau may have direct affects on mitochondrial function and dynamics. As mentioned above, the destabilization of microtubules by mutations in *MAPT* is one way in which mitochondrial transport and synaptic transmission are affected by tau hyperphosphorylation. Most recently, one article reports that overexpression of wild-type tau promotes neurodegeneration in *Drosophila* by blocking mitochondrial localization of Drp1, leading to elongated and dysfunctional mitochondria, and causing neurotoxicity [64]. Though overexpression of wild-type tau is not directly associated with AD pathogenesis, this provides important evidence that tau itself may directly or indirectly influence mitochondrial dynamics both in healthy neurons as well as in AD pathogenesis [65]. In another study, expression of the Asp421-cleaved tau isoform, characteristic in the early stages of AD pathogenesis, has been found to lead to mitochondrial fragmentation in rat neurons [66]. Moreover, another article reported that in SY5Y cells, hyperphosphorylated mutant tau decreases respiratory complex I activity, decreases rates of fission and fusion, and makes cells more vulnerable to oxidative stress [67]. Taken together, these findings suggest that *MAPT* mutations may directly affect mitochondrial function and dynamics, and thereby may constitute a key event in AD pathogenesis by causing a deficit in respiratory complex activity, and by leading to mitochondrial fragmentation, both of which are characteristic of the breakdown of neuronal fitness and activity.

As described above, ER-mitochondria contact sites are critical for maintaining mitochondrial function as well as a diverse range of other cellular activities. Recently, it was discovered that ER-mitochondria contact sites are enriched in the presenilins PS1 and PS2 [68]. Presenilins are aspartyl proteases, which act as the catalytic core in the γ-secretase complex for the proteolytic processing of APP to Aβ [69]. Mutations in PS1- and PS2-encoding genes have been associated with the overproduction of Aβ by γ-secretase and lead to familial AD [70,71]. Interestingly, γ-secretase activity at ER-mitochondria contact sites was found to be approximately five-fold higher than in the ER or mitochondria alone, and thus ER-mitochondria contacts are primary sites of Aβ production [68]. Moreover, new evidence shows that ER-mitochondrial contact sites exhibit increased activity and communication under conditions of AD, resulting in altered calcium signaling and lipid metabolism in affected cells [72]. These data support a new hypothesis in which mutations in genes encoding APP, PS1 and/or PS2 increase Aβ production by γ-secretase at ER-mitochondria contact sites, and thereby alter ER and mitochondrial function as the first step in pathogenesis. As mentioned above, Aβ accumulates in mitochondria, where it induces mtDNA mutations and causes decreased respiratory complex activity [53]. Therefore, under this hypothesis, mutations that lead to increased Aβ production would first impair mitochondrial function, thereby affecting cellular calcium and lipid homeostasis, which may have downstream effects leading to increased Aβ and tau aggregation, and ultimately neuronal death [73]. In this scenario, AD-associated mutations directly impair the function of mitochondria and ER-mitochondria contact sites leading to downstream effects characteristic of AD, thus establishing mitochondria as executors of AD pathogenesis.

### Other neurodegenerative diseases

Since the majority of PD and AD cases are sporadic and not inherited, elucidating the role of mitochondria in neurodegeneration has proved difficult from a genetic standpoint. In some cases, it is difficult to identify if alterations in mitochondrial function are causes of neurodegeneration, or downstream effects of other primary executors. However, in other inheritable neurodegenerative diseases the role of mitochondria as executors of neurodegeneration is more clear, particularly those in which mutations directly affect mitochondrial proteins.

Charcot-Marie-Tooth disease (CMTD) type 2A is an early-onset neurodegenerative disorder, in which mitochondrial dysfunction arising from a nDNA mutation encoding a mitochondrial protein is the primary executor in the onset of neuronal death. CMTD patients carry heterozygous mutations in the gene encoding Mfn2, required for mitochondrial outer membrane fusion [74]. The result is the inhibition of fusion, leading to mitochondrial fragmentation and dysfunction [9]. Moreover, it was recently identified that Mfn2 mutations in CMTD also lead to mtDNA depletion, leading to respiratory complex deficiencies and metabolic dysfunction [75]. Together, this combination of excessive mitochondrial fragmentation and mtDNA depletion lead to severe mitochondrial dysfunction and ultimately the death of peripheral nerves.

Mitochondrial dysfunction associated with inheritable nDNA or mtDNA mutations, which affect mitochondrial function either directly or indirectly, has been implicated in a number of neurodegenerative diseases. Examples include amyotrophic lateral sclerosis, Huntington’s disease, optic atrophy, and spinocerebellar ataxia, the causes of which have been discussed elsewhere [76]. Most recently, mitochondrial dysfunction has been associated with the onset of autism spectrum disorder, providing yet another link between mitochondria and neurological disorders [77].

## Conclusion

Altered mitochondrial function and dynamics are hallmark characteristics of neuronal death. However, whether this dysfunction is a downstream effect of neuropathogenesis, or a key mediator thereof, remains unclear. In the case of PD and CMTD, inheritable disease-associated mutations can directly affect mitochondrial proteins associated with mitochondrial function and dynamics, and therefore provide a more clear genetic linkage between mitochondrial defects and neurodegeneration. In the case of AD, however, the role of mitochondria has been less clear, as mutations carried by AD patients do not directly affect mitochondrial proteins. However, recent findings suggest that mitochondrial dysfunction may precede plaque formation in its pathogenesis, and have implicated Aβ, APP and tau as mediators of mitochondrial function and dynamics, thereby suggesting a link between mitochondrial deficiencies and AD-associated mutations. Taking recent findings into account, alterations in mitochondrial function and dynamics may act as pathomechanisms in neurodegeneration. Therefore, future research should focus on rescuing mitochondrial functional defects, such as respiratory complex activity, as well as restoring the balance of mitochondrial fission and fusion as potential therapeutic targets for neurodegenerative disorders.

## Abbreviations

Aβ: Amyloid β-peptide; AD: Alzheimer’s disease; APP: Amyloid beta precursor protein; ATP: Adenosine triphosphate; CCCP: Carbonyl cyanide *m*-chlorophenylhydrazone; CMTD: Charcot-Marie-Tooth disease; ER: Endoplasmic reticulum; LRRK2: Leucine-rich repeat kinase 2; mtDNA: Mitochondrial DNA; nDNA: Nuclear DNA; PD: Parkinson’s disease; ROS: Reactive oxygen species.

## Competing interests

The author declares that he has no competing interests.

## References

[B1] Nunnari J Suomalainen A Mitochondria: in sickness and in health Cell 2012 148 1145 1159 10.1016/j.cell.2012.02.035 22424226 PMC5381524

[B2] Kazak L Reyes A Holt IJ Minimizing the damage: repair pathways keep mitochondrial DNA intact Nat Rev Mol Cell Biol 2012 13 659 671 10.1038/nrm3439 22992591

[B3] Yang J-L Weissman L Bohr VA Mattson MP Mitochondrial DNA damage and repair in neurodegenerative disorders DNA Repair (Amst) 2008 7 1110 1120 10.1016/j.dnarep.2008.03.012 18463003 PMC2442166

[B4] Zhu X Peng X Guan M-X Yan Q Pathogenic mutations of nuclear genes associated with mitochondrial disorders Acta Biochim Biophys Sin 2009 41 179 187 10.1093/abbs/gmn021 19280056

[B5] Otera H Wang C Cleland MM Setoguchi K Yokota S Youle RJ Mihara K Mff is an essential factor for mitochondrial recruitment of Drp1 during mitochondrial fission in mammalian cells J Cell Biol 2010 191 1141 1158 10.1083/jcb.201007152 21149567 PMC3002033

[B6] Chang C-R Blackstone C Dynamic regulation of mitochondrial fission through modification of the dynamin-related protein Drp1 Ann N Y Acad Sci 2010 1201 34 39 10.1111/j.1749-6632.2010.05629.x 20649536 PMC5585781

[B7] Youle RJ Narendra DP Mechanisms of mitophagy Nat Rev Mol Cell Biol 2011 12 9 14 10.1038/nrm3028 21179058 PMC4780047

[B8] Ding W-X Yin X-M Mitophagy: mechanisms, pathophysiological roles, and analysis Biol Chem 2012 393 547 564 22944659 10.1515/hsz-2012-0119 PMC3630798

[B9] Knott AB Perkins G Schwarzenbacher R Bossy-Wetzel E Mitochondrial fragmentation in neurodegeneration Nat Rev Neurosci 2008 9 505 518 18568013 10.1038/nrn2417 PMC2711514

[B10] Verstreken P Ly CV Venken KJT Koh T-W Zhou Y Bellen HJ Synaptic mitochondria are critical for mobilization of reserve pool vesicles at Drosophila neuromuscular junctions Neuron 2005 47 365 378 10.1016/j.neuron.2005.06.018 16055061

[B11] Chan DC Fusion and fission: interlinked processes critical for mitochondrial health Annu Rev Genet 2012 46 265 287 10.1146/annurev-genet-110410-132529 22934639

[B12] Westermann B Mitochondrial fusion and fission in cell life and death Nat Rev Mol Cell Biol 2010 11 872 884 10.1038/nrm3013 21102612

[B13] Youle RJ van der Bliek AM Mitochondrial fission, fusion, and stress Science 2012 337 1062 1065 10.1126/science.1219855 22936770 PMC4762028

[B14] van Meer G Voelker DR Feigenson GW Membrane lipids: where they are and how they behave Nat Rev Mol Cell Biol 2008 9 112 124 10.1038/nrm2330 18216768 PMC2642958

[B15] Osman C Voelker DR Langer T Making heads or tails of phospholipids in mitochondria J Cell Biol 2011 192 7 16 10.1083/jcb.201006159 21220505 PMC3019561

[B16] Rizzuto R Pinton P Carrington W Fay FS Fogarty KE Lifshitz LM Tuft RA Pozzan T Close contacts with the endoplasmic reticulum as determinants of mitochondrial Ca2+ responses Science 1998 280 1763 1766 10.1126/science.280.5370.1763 9624056

[B17] Friedman JR Lackner LL West M DiBenedetto JR Nunnari J Voeltz GK ER tubules mark sites of mitochondrial division Science 2011 334 358 362 10.1126/science.1207385 21885730 PMC3366560

[B18] Rowland AA Voeltz GK Endoplasmic reticulum-mitochondria contacts: function of the junction Nat Rev Mol Cell Biol 2012 13 607 625 10.1038/nrm3440 22992592 PMC5111635

[B19] Swayne TC Zhou C Boldogh IR Charalel JK McFaline-Figueroa JR Thoms S Yang C Leung G McInnes J Erdmann R Pon LA Role for cER and Mmr1p in anchorage of mitochondria at sites of polarized surface growth in budding yeast Curr Biol 2011 21 1994 1999 10.1016/j.cub.2011.10.019 22119524 PMC3237857

[B20] Kornmann B The molecular hug between the ER and the mitochondria Curr Opin Cell Biol 2013 25 In press 10.1016/j.ceb.2013.02.010 23478213

[B21] Lebiedzinska M Szabadkai G Jones AWE Duszynski J Wieckowski MR Interactions between the endoplasmic reticulum, mitochondria, plasma membrane and other subcellular organelles Int J Biochem Cell Biol 2009 41 1805 1816 10.1016/j.biocel.2009.02.017 19703651

[B22] Bakowski D Nelson C Parekh AB Endoplasmic reticulum-mitochondria coupling: local Ca^2+^ signalling with functional consequences Pflugers Arch 2012 464 27 32 10.1007/s00424-012-1095-x 22415215

[B23] de Lau LML Breteler M Epidemiology of Parkinson’s disease The Lancet Neurology 2006 5 525 535 10.1016/S1474-4422(06)70471-9 16713924

[B24] Hickey P Stacy M Available and emerging treatments for Parkinson’s disease: a review Drug Des Devel Ther 2011 5 241 254 21607020 10.2147/DDDT.S11836 PMC3096539

[B25] Recchia A Debetto P Negro A Guidolin D Skaper SD Giusti P Alpha-synuclein and Parkinson’s disease FASEB J 2004 18 617 626 10.1096/fj.03-0338rev 15054084

[B26] Obeso JA Rodriguez-Oroz MC Goetz CG Marin C Kordower JH Rodriguez M Hirsch EC Farrer M Schapira AHV Halliday G Missing pieces in the Parkinson’s disease puzzle Nat Med 2010 16 653 661 10.1038/nm.2165 20495568

[B27] Kitada T Asakawa S Hattori N Matsumine H Yamamura Y Minoshima S Yokochi M Mizuno Y Shimizu N Mutations in the parkin gene cause autosomal recessive juvenile parkinsonism Nature 1998 392 605 608 10.1038/33416 9560156

[B28] Greene JC Whitworth AJ Kuo I Andrews LA Feany MB Pallanck LJ Mitochondrial pathology and apoptotic muscle degeneration in Drosophila parkin mutants Proc Natl Acad Sci USA 2003 100 4078 4083 10.1073/pnas.0737556100 12642658 PMC153051

[B29] Valente EM Abou-Sleiman PM Caputo V Muqit MMK Harvey K Gispert S Ali Z Del Turco D Bentivoglio AR Healy DG Albanese A Nussbaum R González-Maldonado R Deller T Salvi S Cortelli P Gilks WP Latchman DS Harvey RJ Dallapiccola B Auburger G Wood NW Hereditary early-onset Parkinson’s disease caused by mutations in PINK1 Science 2004 304 1158 1160 10.1126/science.1096284 15087508

[B30] Park J Lee SB Lee S Kim Y Song S Kim S Bae E Kim J Shong M Kim J-M Chung J Mitochondrial dysfunction in Drosophila PINK1 mutants is complemented by parkin Nature 2006 441 1157 1161 10.1038/nature04788 16672980

[B31] Liu G Zhang C Yin J Li X Cheng F Li Y Yang H Uéda K Chan P Yu S alpha-Synuclein is differentially expressed in mitochondria from different rat brain regions and dose-dependently down-regulates complex I activity Neurosci Lett 2009 454 187 192 10.1016/j.neulet.2009.02.056 19429081

[B32] Gui Y-X Wang X-Y Kang W-Y Zhang Y-J Zhang Y Zhou Y Quinn TJ Liu J Chen S-D Extracellular signal-regulated kinase is involved in alpha-synuclein-induced mitochondrial dynamic disorders by regulating dynamin-like protein 1 Neurobiol Aging 2012 33 2841 2854 10.1016/j.neurobiolaging.2012.02.001 22445325

[B33] Narendra DP Jin SM Tanaka A Suen D-F Gautier CA Shen J Cookson MR Youle RJ PINK1 is selectively stabilized on impaired mitochondria to activate Parkin PLoS Biol 2010 8 e1000298 10.1371/journal.pbio.1000298 20126261 PMC2811155

[B34] Abeliovich A Parkinson’s disease: Mitochondrial damage control Nature 2010 463 744 745 10.1038/463744a 20148026

[B35] Schapira AHV Mitochondrial dysfunction in Parkinson’s disease Cell Death Differ 2007 14 1261 1266 10.1038/sj.cdd.4402160 17464321

[B36] Hauser DN Hastings TG Mitochondrial dysfunction and oxidative stress in Parkinson’s disease and monogenic parkinsonism Neurobiol Dis 2013 51 35 42 23064436 10.1016/j.nbd.2012.10.011 PMC3565564

[B37] Yang Y Ouyang Y Yang L Beal MF McQuibban A Vogel H Lu B Pink1 regulates mitochondrial dynamics through interaction with the fission/fusion machinery Proc Natl Acad Sci USA 2008 105 7070 7075 10.1073/pnas.0711845105 18443288 PMC2383971

[B38] Poole AC Thomas RE Andrews LA McBride HM Whitworth AJ Pallanck LJ The PINK1/Parkin pathway regulates mitochondrial morphology Proc Natl Acad Sci USA 2008 105 1638 1643 10.1073/pnas.0709336105 18230723 PMC2234197

[B39] Exner N Treske B Paquet D Holmström K Schiesling C Gispert S Carballo-Carbajal I Berg D Hoepken H-H Gasser T Krüger R Winklhofer KF Vogel F Reichert AS Auburger G Kahle PJ Schmid B Haass C Loss-of-function of human PINK1 results in mitochondrial pathology and can be rescued by parkin J Neurosci 2007 27 12413 12418 10.1523/JNEUROSCI.0719-07.2007 17989306 PMC6673250

[B40] Dagda RK Cherra SJ Kulich SM Tandon A Park D Chu CT Loss of PINK1 function promotes mitophagy through effects on oxidative stress and mitochondrial fission J Biol Chem 2009 284 13843 13855 10.1074/jbc.M808515200 19279012 PMC2679485

[B41] Mata IF Wedemeyer WJ Farrer MJ Taylor JP Gallo KA LRRK2 in Parkinson’s disease: protein domains and functional insights Trends Neurosci 2006 29 286 293 10.1016/j.tins.2006.03.006 16616379

[B42] Wang X Yan MH Fujioka H Liu J Wilson-Delfosse A Chen SG Perry G Casadesus G Zhu X LRRK2 regulates mitochondrial dynamics and function through direct interaction with DLP1 Hum Mol Genet 2012 21 1931 1944 10.1093/hmg/dds003 22228096 PMC3315202

[B43] Wang X Petrie TG Liu Y Liu J Fujioka H Zhu X Parkinson’s disease-associated DJ-1 mutations impair mitochondrial dynamics and cause mitochondrial dysfunction J Neurochem 2012 121 830 839 10.1111/j.1471-4159.2012.07734.x 22428580 PMC3740560

[B44] Ho PW Ho JW Liu H-F So DH Tse ZH Chan K-H Ramsden DB Ho S-L Mitochondrial neuronal uncoupling proteins: a target for potential disease-modification in Parkinson’s disease Transl Neurodegener 2012 1 3 10.1186/2047-9158-1-3 23210978 PMC3506996

[B45] Mayeux R Stern Y Epidemiology of Alzheimer disease Cold Spring Harb Perspect Med 2012 2 1 18 10.1101/cshperspect.a006239 PMC3405821 22908189

[B46] Jack CR Albert MS Knopman DS McKhann GM Sperling RA Carrillo MC Thies B Phelps CH Introduction to the recommendations from the National Institute on Aging-Alzheimer’s Association workgroups on diagnostic guidelines for Alzheimer’s disease Alzheimers Dement 2011 7 257 262 10.1016/j.jalz.2011.03.004 21514247 PMC3096735

[B47] Goedert M Spillantini MG A century of Alzheimer’s disease Science 2006 314 777 781 10.1126/science.1132814 17082447

[B48] Hardy J Selkoe DJ The amyloid hypothesis of Alzheimer’s disease: progress and problems on the road to therapeutics Science 2002 297 353 356 10.1126/science.1072994 12130773

[B49] Rovelet-Lecrux A Hannequin D Raux G Le Meur N Laquerrière A Vital A Dumanchin C Feuillette S Brice A Vercelletto M Dubas F Frebourg T Campion D APP locus duplication causes autosomal dominant early-onset Alzheimer disease with cerebral amyloid angiopathy Nat Genet 2006 38 24 26 10.1038/ng1718 16369530

[B50] Citron M Oltersdorf T Haass C McConlogue L Hung AY Seubert P Vigo-Pelfrey C Lieberburg I Selkoe DJ Mutation of the beta-amyloid precursor protein in familial Alzheimer’s disease increases beta-protein production Nature 1992 360 672 674 10.1038/360672a0 1465129

[B51] Cai XD Golde TE Younkin SG Release of excess amyloid beta protein from a mutant amyloid beta protein precursor Science 1993 259 514 516 10.1126/science.8424174 8424174

[B52] Wisniewski T Ghiso J Frangione B Peptides homologous to the amyloid protein of Alzheimer’s disease containing a glutamine for glutamic acid substitution have accelerated amyloid fibril formation Biochem Biophys Res Commun 1991 180 1528 1953795 10.1016/s0006-291x(05)81370-1

[B53] Manczak M Anekonda TS Henson E Park BS Quinn J Reddy PH Mitochondria are a direct site of A beta accumulation in Alzheimer’s disease neurons: implications for free radical generation and oxidative damage in disease progression Hum Mol Genet 2006 15 1437 1449 10.1093/hmg/ddl066 16551656

[B54] Wang X Su B Fujioka H Zhu X Dynamin-like protein 1 reduction underlies mitochondrial morphology and distribution abnormalities in fibroblasts from sporadic Alzheimer’s disease patients Am J Pathol 2008 173 470 482 10.2353/ajpath.2008.071208 18599615 PMC2475784

[B55] Wang X Su B Siedlak SL Moreira PI Fujioka H Wang Y Casadesus G Zhu X Amyloid-beta overproduction causes abnormal mitochondrial dynamics via differential modulation of mitochondrial fission/fusion proteins Proc Natl Acad Sci USA 2008 105 19318 19323 10.1073/pnas.0804871105 19050078 PMC2614759

[B56] Wang X Su B Lee H-G Li X Perry G Smith MA Zhu X Impaired balance of mitochondrial fission and fusion in Alzheimer’s disease J Neurosci 2009 29 9090 9103 10.1523/JNEUROSCI.1357-09.2009 19605646 PMC2735241

[B57] Calkins MJ Manczak M Mao P Shirendeb U Reddy PH Impaired mitochondrial biogenesis, defective axonal transport of mitochondria, abnormal mitochondrial dynamics and synaptic degeneration in a mouse model of Alzheimer’s disease Hum Mol Genet 2011 20 4515 4529 10.1093/hmg/ddr381 21873260 PMC3209824

[B58] Manczak M Reddy PH Abnormal interaction between the mitochondrial fission protein Drp1 and hyperphosphorylated tau in Alzheimer’s disease neurons: implications for mitochondrial dysfunction and neuronal damage Hum Mol Genet 2012 21 2538 2547 10.1093/hmg/dds072 22367970 PMC3349426

[B59] Hutton M Lendon CL Rizzu P Baker M Froelich S Houlden H Pickering-Brown S Chakraverty S Isaacs A Grover A Hackett J Adamson J Lincoln S Dickson D Davies P Petersen RC Stevens M de Graaff E Wauters E van Baren J Hillebrand M Joosse M Kwon JM Nowotny P Che LK Norton J Morris JC Reed LA Trojanowski J Basun H Association of missense and 5′-splice-site mutations in tau with the inherited dementia FTDP-17 Nature 1998 393 702 705 10.1038/31508 9641683

[B60] Goryunov D Liem RKH CHIP-ping away at tau J Clin Invest 2007 117 590 592 10.1172/JCI31505 17332887 PMC1804367

[B61] Duan Y Dong S Gu F Hu Y Zhao Z Advances in the Pathogenesis of Alzheimer’s Disease: Focusing on Tau-Mediated Neurodegeneration Transl Neurodegener 2012 1 24 10.1186/2047-9158-1-24 23241453 PMC3598890

[B62] Cai Q Sheng Z-H Mitochondrial transport and docking in axons Exp Neurol 2009 218 257 267 10.1016/j.expneurol.2009.03.024 19341731 PMC2710402

[B63] Vos M Lauwers E Verstreken P Synaptic mitochondria in synaptic transmission and organization of vesicle pools in health and disease Front Synaptic Neurosci 2010 2 139 21423525 10.3389/fnsyn.2010.00139 PMC3059669

[B64] DuBoff B Götz J Feany MB Tau promotes neurodegeneration via DRP1 mislocalization in vivo Neuron 2012 75 618 632 10.1016/j.neuron.2012.06.026 22920254 PMC3428596

[B65] DuBoff B Feany M Götz J Why size matters - balancing mitochondrial dynamics in Alzheimer’s disease Trends Neurosci 2013 In press 10.1016/j.tins.2013.03.002 23582339

[B66] Quintanilla RA Dolan PJ Jin YN Johnson GVW Truncated tau and Aβ cooperatively impair mitochondria in primary neurons Neurobiol Aging 2012 33 619 e25–35 21450370 10.1016/j.neurobiolaging.2011.02.007 PMC3140623

[B67] Schulz KL Eckert A Rhein V Mai S Haase W Reichert AS Jendrach M Müller WE Leuner K A new link to mitochondrial impairment in tauopathies Mol Neurobiol 2012 46 205 216 10.1007/s12035-012-8308-3 22847631

[B68] Area-Gomez E de Groof AJC Boldogh I Bird TD Gibson GE Koehler CM Yu WH Duff KE Yaffe MP Pon LA Schon EA Presenilins are enriched in endoplasmic reticulum membranes associated with mitochondria Am J Pathol 2009 175 1810 1816 10.2353/ajpath.2009.090219 19834068 PMC2774047

[B69] Takasugi N Tomita T Hayashi I Tsuruoka M Niimura M Takahashi Y Thinakaran G Iwatsubo T The role of presenilin cofactors in the gamma-secretase complex Nature 2003 422 438 441 10.1038/nature01506 12660785

[B70] Citron M Westaway D Xia W Carlson G Diehl T Levesque G Johnson-Wood K Lee M Seubert P Davis A Kholodenko D Motter R Sherrington R Perry B Yao H Strome R Lieberburg I Rommens J Kim S Schenk D Fraser P St George Hyslop P Selkoe DJ Mutant presenilins of Alzheimer’s disease increase production of 42-residue amyloid beta-protein in both transfected cells and transgenic mice Nat Med 1997 3 67 72 10.1038/nm0197-67 8986743

[B71] Tomita T Maruyama K Saido TC Kume H Shinozaki K Tokuhiro S Capell A Walter J Grünberg J Haass C Iwatsubo T Obata K The presenilin 2 mutation (N141I) linked to familial Alzheimer disease (Volga German families) increases the secretion of amyloid beta protein ending at the 42nd (or 43rd) residue Proc Natl Acad Sci USA 1997 94 2025 2030 10.1073/pnas.94.5.2025 9050898 PMC20036

[B72] Area-Gomez E del Carmen Lara Castillo M Tambini MD Guardia-Laguarta C de Groof AJC Madra M Ikenouchi J Umeda M Bird TD Sturley SL Schon EA Upregulated function of mitochondria-associated ER membranes in Alzheimer disease EMBO J 2012 31 4106 4123 10.1038/emboj.2012.202 22892566 PMC3492725

[B73] Schon EA Area-Gomez E Mitochondria-associated ER membranes in Alzheimer disease Mol Cell Neurosci 2012 55 26 36 22922446 10.1016/j.mcn.2012.07.011

[B74] Züchner S Mersiyanova IV Muglia M Bissar-Tadmouri N Rochelle J Dadali EL Zappia M Nelis E Patitucci A Senderek J Parman Y Evgrafov O Jonghe PD Takahashi Y Tsuji S Pericak-Vance MA Quattrone A Battaloglu E Polyakov AV Timmerman V Schröder JM Vance JM Battologlu E Mutations in the mitochondrial GTPase mitofusin 2 cause Charcot-Marie-Tooth neuropathy type 2A Nat Genet 2004 36 449 451 10.1038/ng1341 15064763

[B75] Vielhaber S Debska-Vielhaber G Peeva V Schoeler S Kudin AP Minin I Schreiber S Dengler R Kollewe K Zuschratter W Kornblum C Zsurka G Kunz WS Mitofusin 2 mutations affect mitochondrial function by mitochondrial DNA depletion Acta Neuropathol 2013 125 245 256 10.1007/s00401-012-1036-y 22926664

[B76] Schon EA Przedborski S Mitochondria: the next (neurode)generation Neuron 2011 70 1033 1053 10.1016/j.neuron.2011.06.003 21689593 PMC3407575

[B77] Tang G Gutierrez Rios P Kuo S-H Akman HO Rosoklija G Tanji K Dwork A Schon EA DiMauro S Goldman J Sulzer D Mitochondrial abnormalities in temporal lobe of autistic brain Neurobiol Dis 2013 54 349 361 23333625 10.1016/j.nbd.2013.01.006 PMC3959772

